# Sustained TNF production by central nervous system infiltrating macrophages promotes progressive autoimmune encephalomyelitis

**DOI:** 10.1186/s12974-016-0513-y

**Published:** 2016-02-22

**Authors:** Alice Valentin-Torres, Carine Savarin, David R. Hinton, Timothy W. Phares, Cornelia C. Bergmann, Stephen A. Stohlman

**Affiliations:** Department of Neurosciences NC-30, Lerner Research Institute, The Cleveland Clinic, 9500 Euclid Ave., Cleveland, OH 44195 USA; Department of Pathology, Keck School of Medicine, University of Southern California, Los Angeles, CA 90033 USA; Malaria Vaccine Branch, Walter Reed Army Institute of Research, Silver Spring, MD 20910 USA

**Keywords:** Progressive multiple sclerosis, Experimental autoimmune encephalomyelitis, Astrocytes, Interferon γ, Tumor necrosis factor

## Abstract

**Background:**

Tumor necrosis factor (TNF) has pleiotropic functions during both the demyelinating autoimmune disease multiple sclerosis (MS) and its murine model experimental autoimmune encephalomyelitis (EAE). How TNF regulates disability during progressive disease remains unresolved. Using a progressive EAE model characterized by sustained TNF and increasing morbidity, this study evaluates the role of unregulated TNF in exacerbating central nervous system (CNS) pathology and inflammation.

**Methods:**

Progressive MS was mimicked by myelin oligodendrocyte glycoprotein (MOG) peptide immunization of mice expressing a dominant negative IFN-γ receptor alpha chain under the human glial fibrillary acidic protein promoter (GFAPγR1∆). Diseased GFAPγR1∆ mice were treated with anti-TNF or control monoclonal antibody during acute disease to monitor therapeutic effects on sustained disability, demyelination, CNS inflammation, and blood brain barrier (BBB) permeability.

**Results:**

TNF was specifically sustained in infiltrating macrophages. Anti-TNF treatment decreased established clinical disability and mortality rate within 7 days. Control of disease progression was associated with a decline in myelin loss and leukocyte infiltration, as well as macrophage activation. In addition to mitigating CNS inflammation, TNF neutralization restored BBB integrity and enhanced CNS anti-inflammatory responses.

**Conclusions:**

Sustained TNF production by infiltrating macrophages associated with progressive EAE exacerbates disease severity by promoting inflammation and disruption of BBB integrity, thereby counteracting establishment of an anti-inflammatory environment required for disease remission.

## Background

Tumor necrosis factor (TNF) is a pleiotropic cytokine that regulates numerous physiological and pathological processes. Specifically, the pathophysiology of a variety of neurological disorders, including the demyelinating autoimmune disease multiple sclerosis (MS) is associated with TNF [1]. Expression of TNF in active MS lesions [2], as well as elevated TNF in serum and cerebral spinal fluid, correlates with lesion activity [3]. Increased TNF in the central nervous system (CNS) of animals undergoing experimental autoimmune encephalomyelitis (EAE), a rodent model of MS, supports pro-inflammatory and disease enhancing activity. Interestingly, increased TNF in spinal cords also coincides with neuropathic pain in rats undergoing EAE [4]. Furthermore, transgenic expression of TNF within the CNS leads to demyelinating disease [5–7]. This is consistent with the ability of TNF to both directly induce oligodendrocyte apoptosis [8–10] and indirectly inflict excitotoxic damage to oligodendrocytes and neurons by modulating the release of glutamate from astrocytes [8, 11]. A pro-inflammatory role of TNF in CNS autoimmune disease is further supported by the observations that TNF blockade prior to disease onset prevents or ameliorates EAE [12, 13]. The compelling evidence suggesting destructive roles for TNF in MS patients, EAE, in vitro studies, and other autoimmune diseases provided the basis to target TNF to treat MS patients. Infliximab, a mouse/human chimeric monoclonal anti-TNF antibody, was used in a first open-label phase trial I on two rapidly progressing MS patients [14]. Both patients showed increased lesion numbers, leukocytes in cerebrospinal fluid, and IgG titers correlating with an augmentation in disease activity. Moreover, a randomized Phase II placebo-controlled trial with Lenercept, an extracellular domain of dimeric TNFR1 fused with IgG1 heavy chain fragment, was conducted in relapsing-remitting MS patients. However, the trial was abruptly terminated due to a dose-dependent increase in frequency and severity of MS attacks [15].

EAE has provided insights into the dual pro- and anti-inflammatory activities exhibited by TNF during CNS autoimmunity. Two forms of TNF, a soluble and transmembrane form, interact with two distinct receptors, namely TNF receptor 1 (TNFR1) and TNF receptor 2 (TNFR2), which differ in expression and ligand affinity. The pleiotropic functions of TNF are predominantly dictated by interaction with these two receptors. Soluble TNF with higher affinity for TNFR1 mediates apoptosis and chronic inflammation [16]. Conversely, transmembrane TNF with higher affinity for TNFR2 activates genes important for cell survival, resolution of inflammation, and even myelination [17–19]. Consistent with these concepts, both TNF^−/−^ and TNFR2^−/−^ mice develop more severe EAE [17, 20, 21], while TNFR1^−/−^ mice are protected from EAE [20]. The absence of both TNFR1 and TNFR2 delays disease onset but does not protect [20]. Furthermore, mice expressing only transmembrane TNF demonstrated its protective role during EAE [17]. A recent pharmacological approach eliminating only soluble TNF without inhibiting transmembrane TNF supported the concept that inhibition of soluble TNF is therapeutic during EAE [22]. Interestingly, following inhibition of soluble TNF, recovery from paralysis was more rapid and associated with increased axonal preservation and remyelination [22]. Similar to the analysis of EAE in TNF^−/−^ mice [17, 20, 21], inhibition of both soluble TNF and transmembrane TNF were not protective [13, 22, 23].

Collectively, these studies suggest that the protective effects of TNF in EAE are mediated by the interaction of transmembrane TNF with TNFR2 and that blocking these anti-inflammatory properties during the relapsing-remitting phase of MS contributes to the adverse outcome. Contrasting these studies predominantly focusing on the effect of TNF on either disease induction or the acute phase of EAE, the study herein specifically addresses the role of TNF during a chronic, non-remitting form of EAE. We have previously reported that mice, in which astrocytes are unable to respond to interferon gamma (IFN-γ), develop a chronic progressive form of EAE [24] with many pathophysiological hallmarks of progressive MS. Increasing clinical disease and mortality in this model are associated with sustained inflammation, sustained interleukin-6 (IL-6) secretion [25], increased TNF [24], and increased myelin destruction [24, 25]. Blockade of IL-6, associated with a variety of neurological dysfunctions [25–27], was therapeutic by reducing clinical disease, inflammation, and demyelination [25]. Based on the ability of TNF to induce IL-6 secretion by astrocytes [28–30] and the reduction in secondary progressive disease in MS patients treated with a TNF inhibitor [31, 32], we examined the therapeutic potential of inhibiting both soluble and transmembrane TNF in the progressive EAE model. Comparison of acute EAE in both wild-type (WT) and GFAPγR1∆ mice confirmed that TNF is predominantly produced by both macrophages and microglia. However, while TNF levels decreased in both populations in WT mice during disease remission, TNF was specifically sustained by infiltrating macrophages during progressive EAE. Although blockade of TNF did not promote remission in WT mice, it significantly ameliorated clinical symptoms and decreased mortality during progressive EAE. Reduced clinical disease following TNF blockade was associated with decreased CNS macrophage and T cells accumulation, including both Th1 and Th17 cells, as well as reduced microglial and macrophage activation. Anti-TNF therapy further restored blood brain barrier (BBB) integrity and promoted an anti-inflammatory milieu consistent with diminished tissue damage. Overall, the data support distinct regulation of macrophages and microglia during progressive disease, with sustained TNF production in macrophages contributing to clinical disability and CNS inflammation. Amelioration of progressive demyelinating encephalomyelitis by TNF blockade supports the concept that TNF is a potential target for therapeutic approaches in both primary and secondary progressive MS.

## Methods

### Mice

Homozygous H-2^b^ GFAP/IFN-γR1∆IC (GFAPγR1∆) transgenic mice expressing a dominant negative IFN-γ receptor alpha chain under control of the glial fibrillary acidic protein (GFAP) promoter [24, 33] were bred locally. C57BL/6 (H-2^b^) WT mice were purchased from the National Cancer Institute (Frederick, MD). FVB/N-Tg (GFAP-GFP) 14Mes/J mice (The Jackson Laboratory, Bar Harbor, ME) were backcrossed 11 times into the C57BL/6 background to generate C57BL/6 WT mice expressing green fluorescent protein (GFP) under the GFAP promoter (GFAP-GFP). GFAP-GFP mice were crossed with GFAPγR1∆ mice to generate dual transgenic GFAP-GFP/GFAPγR1∆ mice. All animal experiments were conducted in mice from both sexes in accordance with the National Institute of Health Guide for the Care and Use of Laboratory Animals. All procedures were performed in compliance with protocols (Protocol number 1165) approved by the Cleveland Clinic Institutional Animal Care and Use committee.

### EAE

EAE was induced by immunizing mice with myelin oligodendrocyte glycoprotein (MOG)^35–55^ peptide (Biosynthesis, Lewisville, TX) emulsified at 3 mg/ml in PBS with an equal amount of incomplete Freund’s Adjuvant (IFA; Sigma-Aldrich, St. Louis, MO) supplemented with 5 mg/ml of *Mycobacterium tuberculosis*, strain H37Ra (Difco, Detroit, MI). Mice were immunized subcutaneously with 200 μl emulsion over the flanks. Mice also received 200 ng of Pertussis toxin (Sigma-Aldrich) in 200 μl of PBS intraperitoneally (i.p.) on the day of the initial immunization and at day 2 post-immunization (p.i.). Mice received a second immunization with 200 μl emulsion in the left side over the flanks 7 days later. Animals were scored daily for clinical symptoms as follows: 0 = no signs of disease, 1 = flaccid tail, 2 = flaccid tail and partial hind limb paralysis, 3 = complete hind limb paralysis, 4 = moribund state, 5 = dead.

### Astrocyte and microglia purification by cell sorting

EAE was induced in GFAP-GFP/GFAPγR1∆ as described above. At d19 and d30 p.i., brains were homogenized using a Papain Neuronal Tissue Dissociation Kit (Miltenyi, Auburn CA). Cells from homogenates were resuspended in RPMI medium containing 25 mM HEPES (pH 7.2) and adjusted to 30 % Percoll (Amersham Bioscience, Piscataway, NJ). A 1-ml underlay of 70 % Percoll was added prior to centrifugation at 800×*g* for 30 min at 4 °C to separate mononuclear cells from myelin debris. Cells from the 30/70 % interface were collected and washed in RPMI medium. Antibody non-specific binding was prevented with mouse anti-CD16/CD32 (2.4G2; BD Biosciences, San Diego, CA) and 10 % mixture of normal goat, human, mouse, and rat serum for 10 min on ice. To distinguish astrocytes from microglia and inflammatory cells, recovered cells were stained with anti-CD45 (clone 30-F11) APC and CD11b (M1/70) PerCp (BD Biosciences, San Diego, CA). Astrocytes were identified as GFAP-GFP^+^ CD45^-^, whereas microglia were defined as CD45^int^ CD11b^+^. Cells were purified by FACS using a FACSAria (BD Biosciences) and FACS Diva software (BD Biosciences).

### Anti-TNF treatment

Mice received 0.5 mg of either neutralizing rat anti-murine TNF (clone MD6-XT3.11) or control rat IgG1 anti-β-galactosidase (clone GL113) monoclonal antibody (mAb) i.p. starting at the peak of acute disease (day 19 p.i.) followed by injections every 2 days for a total of four dosages. Anti-TNF mAb was purchased from Bioxcell, West Lebanon, NH. The GL-113 hybridoma, originally obtained from Dr. Robert Coffman (DNAX Corp, Palo Alto, CA) was adapted to BD cell Serum Free Medium (BD, Bedford, MA). After propagation in a BD CELLine device, the mAb concentration was determined by optical density at 480 nm. All mAbs were diluted to 1 mg/ml in endotoxin free PBS and stored at −20 °C until use.

### Flow cytometry

Brains from mice perfused with ice-cold PBS were individually homogenized in 4 ml of RPMI medium containing 25 mM HEPES, pH 7.2 using chilled Tenbroeck tissue grinders. Homogenates were centrifuged at 450×*g* for 7 min. Supernatants were collected and immediately stored at −80 °C for cytokine analysis (see below). Cells were resuspended in 30 % Percoll and isolated by centrifugation (800×*g* for 30 min at 4 °C) onto a 70 % Percoll cushion as described above for cell sorting. Non-specific antibody binding was inhibited by incubation with anti-CD16/CD32 (2.4G2 mAb) and a 10 % mixture of normal goat, human, mouse, and rat serum for 15 min on ice. CD45 (30-F11) APC, CD4 (GK1.5) PercP-Cy5.5, CD8a (53−6.7) FITC, and CD11b (M1/70) PE mAbs (BD Biosciences) were used to analyze microglia and infiltrating inflammatory cells (BD Biosciences). Microglia and inflammatory cells were distinguished based on their differential CD45 expression. Major histocompatibility complex (MHC) class II (clone 2G9, BD Biosciences) expression was determined as a measure of macrophage and microglial activation. To detect intracellular cytokines cells were stimulated ex vivo for a total of 4 h at 37 °C with phorbol 12-myristate 13-acetate (PMA) (10 ng/ml, Acros Organics, NJ) and ionomycin (1 μM, Calbiochem, Spring Valley, CA). Monensin (2 μM, Calbiochem) was added for the last 2 h of incubation. Following permeabilization (Cytofix/Cytoperm Reagent, BD Biosciences) intracellular cytokines were detected using anti-IFN-γ (clone XMG1.2; BD Bioscience) and anti-IL-17 (clone TC11-18H10; BD Bioscience) mAb. To determine intracellular TNF, cells were incubated in serum free RPMI at 37 °C for 4 h with Monensin (2 μM) added for the last 2 h. After 4 h, cells were permeabilized with cytofix/cytoperm reagent (BD Biosciences) and stained using PE anti-TNF mAb (clone MP6-XT22; BD, Bioscience).

### ELISA and BBB permeability

IFN-γ and IL-17 were determined by ELISA as previously described [34] using mAb pairs and recombinant cytokine standards from BD Bioscience. IL-10 was measured by ELISA using reagents from eBiosciences, San Diego, CA.

BBB integrity was determined by measuring permeability to sodium fluorescein (NaF) as described [35]. Mice received 100 μl of 10 % NaF in PBS i.p. After 10 min, cardiac blood was collected, followed by transcardial perfusion with ice-cold PBS. Brains and spinal cords were harvested and snap-frozen in liquid nitrogen. Each tissue was weighed and homogenized in 10 ml of PBS per gram of tissue followed by centrifugation for 2 min at 14,000×*g* at 4 °C. Supernatants were mixed with an equal volume of 15 % trichloroacetic acid and centrifuged at 10,000×*g* for 10 min at 4 °C. After centrifugation, 125 μl of 5 N NaOH was added to 500 μl of supernatant. NaF concentrations were determined by comparison to standards ranging from 125 to 4000 μg on a SpectraMax M2 at an excitation of 485 nm, emission of 530 nm, and gain of 50 (Molecular Devices, Sunny Valley, CA). CNS values were normalized to NaF serum levels as follows: (μg florescence tissue/mg protein)/(μg fluorescence sera/μl of blood) [35]. Data are presented as fold increase by comparing experimental levels to those obtained from naïve WT mice with intact BBB.

### Immunohistochemistry and histopathology

Spinal cords from WT and GFAPγR1∆ mice perfused with ice-cold PBS during acute and chronic EAE were collected in OCT embedding compound (Scigen Scientific, Gardena CA). Frozen sections were stained with anti-mouse TNF (1:100) (Abcam Inc., Cambridge, MA), anti-mouse Iba1 (1:200) (Wako Chemicals, Richmond, VA) or anti-mouse GFAP (1:5000) (Dako, Carpinteria, CA), and Prolong Gold for nuclear staining (Molecular Probes, Eugene, OR) as previously shown [36]. Immunoreactivity was visualized with species-specific fluorescently labeled secondary antibodies (goat anti-rabbit Alexa Fluor 488, Life Technologies Carlsbad, CA; goat anti-mouse Alexa Fluor 594, Invitrogen, Grand Island, NY). Images were captured using a multiphoton microscope (Leica TCS SP5 II, Lawrenceville, GA). TNF^+^ area was calculated as an average of three different fields per group using Image-Pro.

Spinal cord frozen sections were also stained with laminin (1:2000) (Abcam Inc., Cambridge, MA), IgG conjugated with Alexa Fluor-594 (1:200) (Jackson Laboratories, Sacramento CA), and Prolong Gold. Laminin was visualized using goat anti-rabbit Alexa Fluor-488 (Life Technologies).

Spinal cords were obtained from mice perfused with ice-cold PBS and fixed in Zn Formalin. Each spinal cord was dissected into six sections, two of each corresponding to the cervical, thoracic, and lumbar regions and embedded in paraffin. To determine the percentage demyelination, 6-μm cross sections were stained with Luxol Fast Blue (LFB), scanned at 40× and digitally imaged at high resolution with an Aperio Scanscope (Vista, CA) as described [25]. Aperio software was used to quantify areas of demyelination within the white matter tracks.

### Gene expression analysis

Following PBS perfusion, snap-frozen spinal cords were homogenized in Trizol (Invitrogen) using a TissueLyser with stainless beads (Qiagen, Valencia, CA). RNA was extracted according to the manufacturer’s instructions followed by DNAse I (Ambion, Austin, TX) treatment for 30 min at 37 °C. M-MLV Reverse Transcriptase (Invitrogen), oligo-dT primers (20 μM) (Promega, Madison, WI), and random primers (20 μM) (Promega) were used to synthesize cDNA. Gene expression analysis was performed by quantitative real-time PCR using a 7500 Fast real-time PCR system (Applied Biosystems, Foster City, CA). Foxp3 and IL-27 p28 messenger RNA (mRNA) levels were detected by Taqman Gene Expression Assay using Taqman primers (Foxp3 -Mm00475162_m1, IL-27 p28-Mm00461162_m1) (Life Technologies, Carlsbad, CA).

RNA was extracted from FACS-purified cell populations frozen in 400 μl of Trizol reagent (Invitrogen, Waltham, MA). Removal of DNA contamination and cDNA synthesis were performed as described above. TNF mRNA expression analysis was determined using 4 μl of cDNA and SYBR Green master mix (Applied Biosystems, Foster City, CA) containing TNF primers (GCCACCACGCTCTTCTGTCT and GGTCTGGGCCATAGAACTGATG).

### Statistical analysis

Data represent the mean ± SEM. Significance was determined using two-tailed Student’s *t* test or Mann-Whitney Rank Sum test using SigmaStat v3.5. A value of *p* < 0.05 was considered statistically significant. Graphs were plotted using GraphPad Prism v5.02 software.

## Results

### TNF production is sustained in macrophages during progressive EAE

**Fig. 1:**
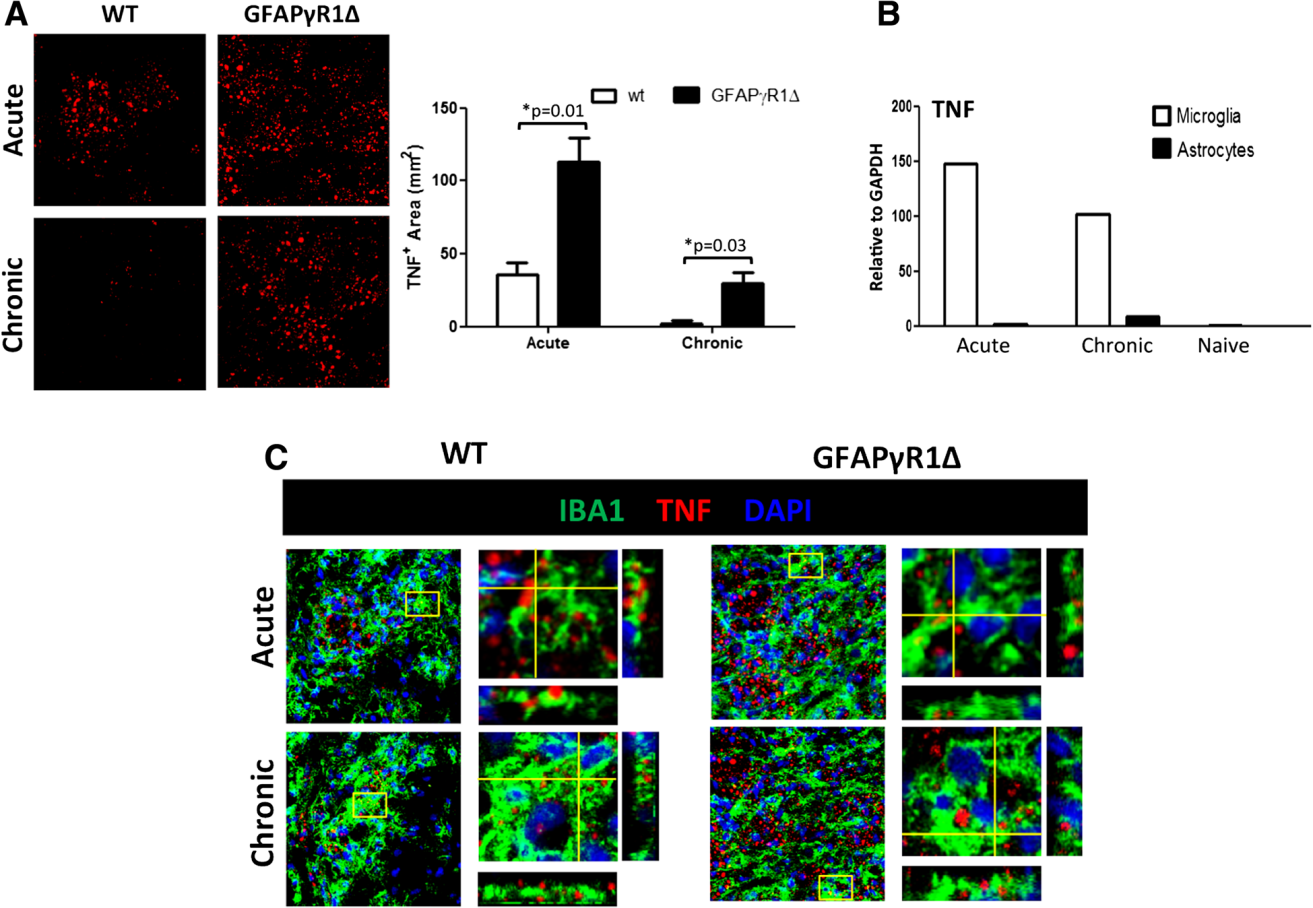
TNF is primarily produced by Iba1^+^ cells during EAE. **a** Confocal Z-stack images showing TNF in white matter of spinal cords from WT and GFAP∆R1γ during acute and chronic EAE. Magnification ×100. *Bar graph* depicts TNF^+^ area calculated as an average of three fields per group. **b** TNF mRNA expression in FACS sorted astrocytes and microglia from GFAP-GFP/GFAP∆R1γ during acute (d19 p.i.) and chronic EAE (d30 p.i.). **c** Confocal Z-stack images (magnification ×100) with orthogonal views illustrating TNF co-staining with Iba1 in spinal cord white matter from WT and GFAP∆R1γ during acute and chronic EAE. Data are representative of three mice per group. *P* values determined by Student’s *t* test

In active MS lesions, TNF is produced by microglia, macrophages, and astrocytes, but not T cells [2]. Similarly, analysis of acute EAE in a relapsing/remitting model indicated that TNF is primarily produced by microglia and/or bone marrow derived macrophages with little or no expression by T cells [37]. Elevated TNF mRNA expression in the CNS is also a feature distinguishing acute and progressive EAE in GFAP∆R1γ compared to WT mice [33], although the primary source is unknown. To first verify that TNF protein production in the CNS reflects differences in mRNA, TNF expression was monitored by histology in spinal cords of WT and GFAP∆R1γ mice (Fig. 1a). Naïve mice did not express detectable TNF (data not shown). In mice undergoing acute EAE, TNF was readily detected in white matter of both groups with an overall similar distribution and focal clustering (Fig. 1a). Focal clustering of anti-TNF reactivity was also present in gray matter, albeit less intense (data not shown). TNF mRNA declines during the recovery phase of EAE in WT mice [38, 39], which is characterized by sustained demyelination with minimal active inflammation [39]. By contrast, increasing morbidity during progressive EAE is associated with sustained TNF mRNA expression [24]. This pattern was also reflected by histological analysis. Contrasting the minimal TNF production during the recovery phase in WT mice, TNF was sustained in the CNS of mice with progressive EAE, although at lower levels compared to the acute phase (Fig. 1a). Irrespectively, the overall punctate, constricted nature of TNF mAb reactivity was similar between groups.

We next examined the cell types associated with TNF production. As astrocytes can be a potential source of TNF [2, 40, 41], we specifically assessed if IFN-γ regulates TNF in astrocytes. Comparative analysis of TNF mRNA in astrocytes and microglia purified from dual transgenic GFAP∆R1γ/GFAP-GFP mice revealed prominent TNF mRNA levels in microglia during both acute and chronic EAE (Fig. 1b). By contrast, astrocytes showed minimal TNF mRNA upregulation compared to naïve mice. There was also no evidence of TNF co-localization with either GFAP^+^ astrocytes or T cells by histology during either the acute or chronic phase in either mouse group (data not shown). TNF primarily co-localized with Iba1^+^ cells in both groups of mice undergoing acute EAE (Fig. 1c). The sparse CNS cells producing TNF during the recovery phase in WT mice were also Iba1^+^ (Fig. 1c). Similarly, although the numbers were higher during progressive EAE, TNF was primarily associated with Ibal^+^ cells. As the overlap between Iba1 and TNF staining was limited, side view images are shown in Fig. 1c to confirm juxtaposition of TNF to Iba1^+^ membranes and containment within Iba1^+^ cells. The punctate TNF staining pattern was consistent with vesicle cytosolic associated membrane TNF, rather than dispersed surface associated TNF [42], implicating TNF was cleaved once fused with the cell membrane. These results confirmed macrophages and/or microglia as prominent sources of TNF, independent of IFN-γ signaling to astrocytes, and are consistent with data from the acute phase in a relapsing/remitting model of EAE [37].

**Fig. 2:**
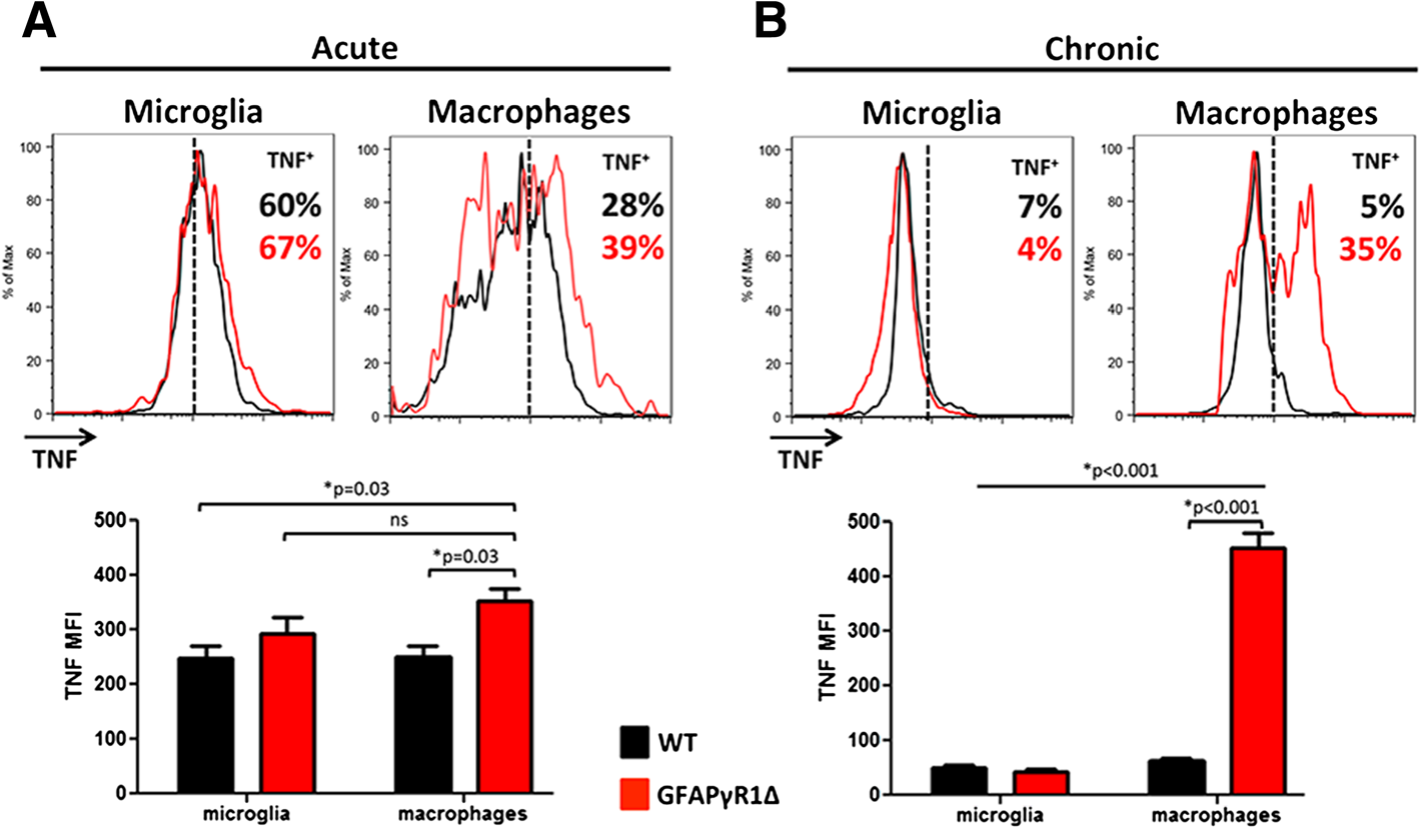
TNF secretion is sustained in infiltrating macrophages during progressive EAE. TNF production by macrophages and microglia determined by intracellular staining during **a** acute (d19 p.i.) and **b** chronic (d30 p.i.) EAE in WT (*black line*) or GFAP∆R1γ (*red line*) mice. *Numbers in histograms* depict the percentage of TNF producing WT cells (*black*) or GFAP∆R1γ cells (*red*). *Dotted line* represents isotype control. *Bar graphs* show mean fluorescence intensity (MFI) of TNF staining in macrophages and microglia from WT and GFAP∆R1γ mice during acute and chronic disease. Data represent the mean values and ± SEM of three independent experiments with six mice per group per experiment. *P* values determined by Student’s *t* test

Flow cytometry was used to further distinguish between Iba1^+^ CD45^low^ microglia and CNS infiltrating CD45^hi^ CD11b^+^ macrophages as the predominant source of TNF. The majority (~70 %) of microglia produced TNF during acute EAE in both mouse groups, with only a minor increase at the population level evident by mean fluorescent intensity (MFI) in GFAP∆R1γ mice (Fig. 2a). By contrast, although only a third of CNS infiltrating macrophages produced TNF in either group, increased TNF production per cell in macrophages from GFAP∆R1γ mice was supported by increased MFI. During the remission phase in WT mice, the proportion of cells producing TNF dropped to less than 7 % for both macrophages and microglia (Fig. 2b). Moreover, the MFI was extremely low (Fig. 2b). Similarly, during progressive EAE, both the proportion and MFI of microglia producing TNF were reduced (Fig. 2b). By contrast, TNF secretion was maintained in ~40 % of macrophages with MFI at nearly the levels expressed during the acute phase (Fig. 2b), distinct from the paucity of TNF producing macrophages in remitting WT mice. These data are consistent with mRNA results suggesting minimal TNF production during the chronic/repair phase of EAE in WT mice [38, 39]. Moreover, they provide the first evidence that TNF is down regulated in both microglia and macrophages during remission of EAE. By contrast, despite the ~50 % reduction in infiltrating macrophages during the progressive phase relative to acute EAE [24], macrophages sustained TNF expression in GFAP∆R1γ mice. The loss of microglia but not macrophages secreting TNF, suggests differential regulation of both populations in the same environment. The progressive phase does therefore not merely reflect a sustained acute phase.

### TNF blockade limits disease progression and demyelination during progressive EAE

**Fig. 3:**
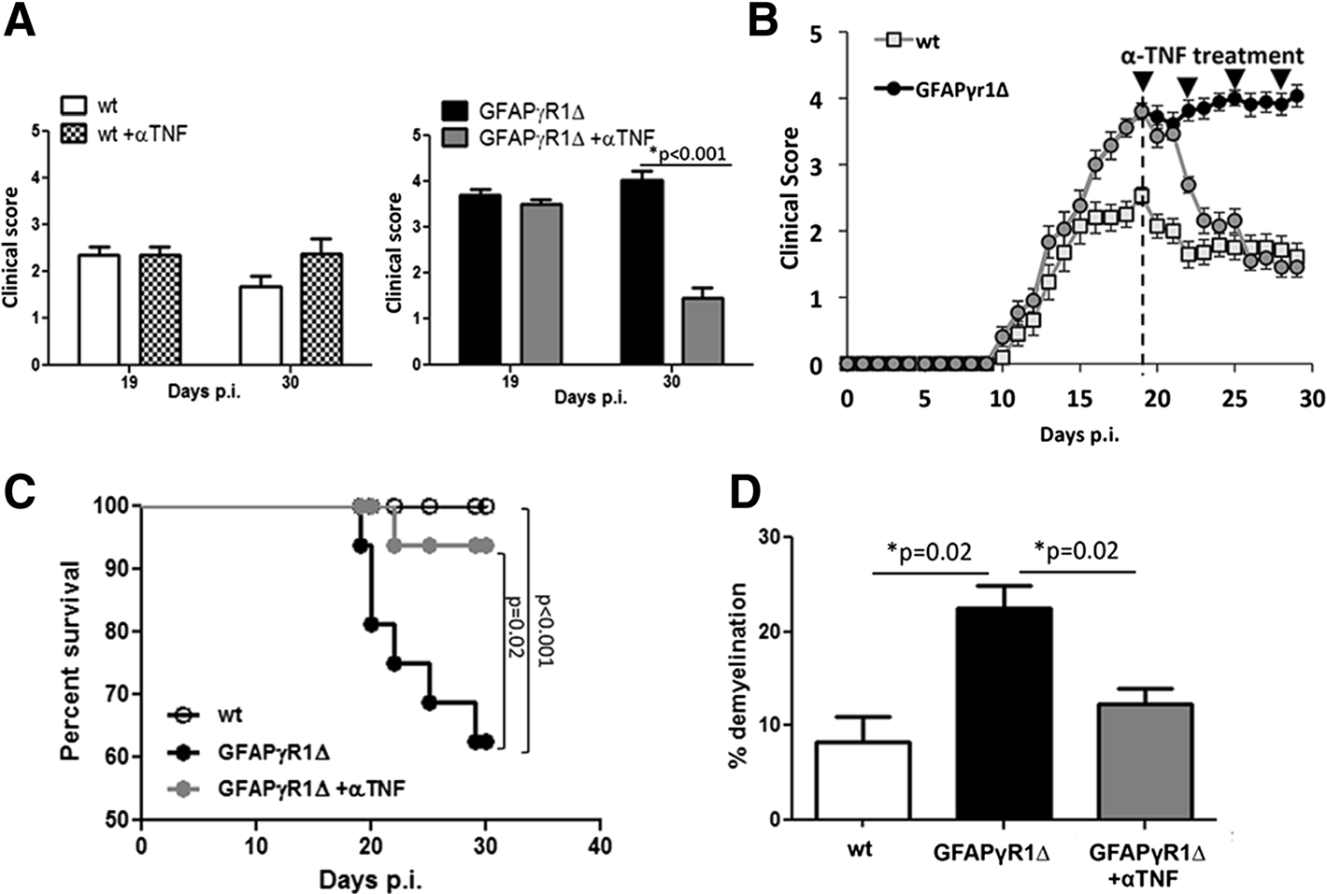
Anti-TNF treatment ameliorates progressive EAE. EAE was induced in WT and GFAP∆R1γ mice. At the peak of acute disease (d19 p.i.), WT and GFAP∆R1γ were divided into two groups with equal clinical scores. WT + αTNF and GFAP∆R1γ + αTNF received i.p. injections of anti-TNF mAb (0.5 mg), whereas WT and GFAP∆R1γ received equal amounts of isotype mAb control. Mice were treated every 2 days for a total of four dosages. **a** Clinical scores of αTNF treated and control cohorts pre and 10 days post treatment **b** Kinetics of clinical recovery and **c** percent survival in anti-TNF treated GFAP∆R1γ mice relative to control treated GFAP∆R1γ and WT mice. Data represent the mean values and ± SEM of four separate experiments with five mice per group per experiment. *P* values are determined by Student’s *t* test. **d** Percentage area of demyelination in spinal cord white matter calculated by analysis of transverse sections at six separate levels per mouse. Data represent the mean ± SEM of seven to nine individual mice per group. Statistical differences determined by two-tailed unpaired *t* test with *p < 0.05

To determine if sustained macrophage derived TNF contributes to progressive EAE, GFAPγR1∆ and WT mice undergoing acute disease were each divided into two cohorts (Fig. 3a). The cohorts were treated with either anti-TNF mAb neutralizing both soluble and transmembrane TNF or an isotype control mAb. TNF blockade in WT mice had little effect on clinical symptoms during EAE recovery (Fig. 3a), consistent with previous data [13, 22, 23]. By contrast, TNF blockade significantly improved clinical disease within 10 days in GFAPγR1∆ mice (Fig. 3a). Direct comparison of clinical scores to recovering WT mice already revealed dramatic clinical improvement of GFAPγR1∆ mice within 6 days after two anti-TNF treatments, while disease continued to deteriorate in the control cohort (Fig. 3b). In addition to limiting disease progression, TNF blockade also significantly reduced mortality in GFAPγR1∆ mice (Fig. 3c).

During progressive EAE, GFAPγR1∆ mice exhibit increased demyelination compared to WT mice [24]. To determine to what extent clinical improvement by TNF blockade is associated with limited demyelination, we evaluated demyelination in spinal cords of GFAPγR1∆ mice treated with anti-TNF compared to GFAPγR1∆ controls and WT mice. Anti-TNF treatment reduced demyelination in the GFAPγR1∆ mice to approximately the level found in WT mice (Fig. 3d). Quantitative analysis of demyelination in white matter revealed that ~23 % of white matter area was affected in control treated-GFAPγR1∆ mice compared to only ~8 % in WT mice, consistent with previous data [24, 25]. TNF blockade reduced demyelination by 46 to ~12 % of white matter area, approximating the extent of demyelination in WT mice. Irrespective of the severe pathology prior to treatment during acute EAE in GFAPγR1∆ mice, clinical improvement thus correlated with limited demyelination similar to levels observed in untreated WT mice (Fig. 3b).

### TNF blockade limits inflammation indiscriminately during progressive EAE

**Fig. 4:**
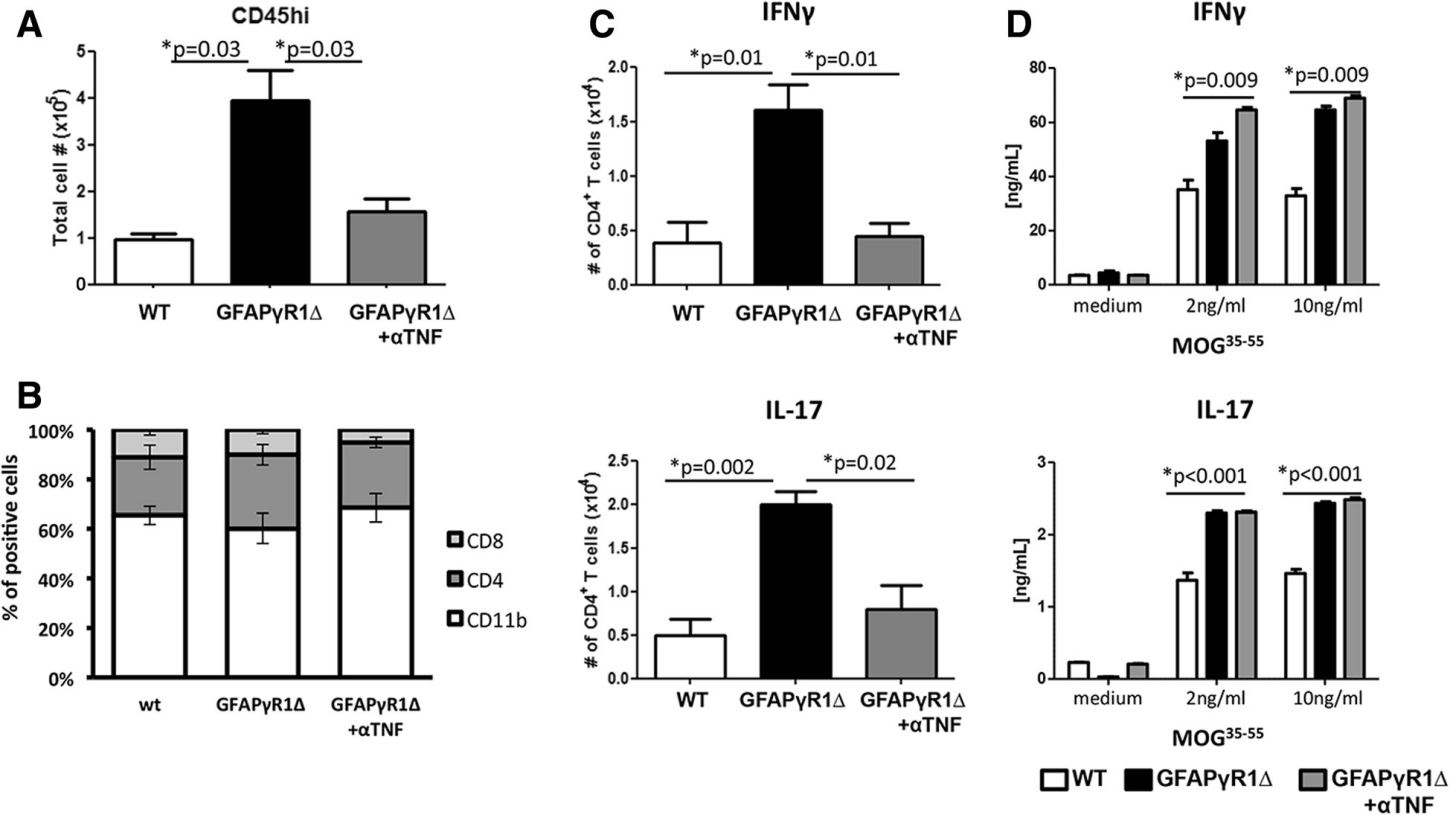
Anti-TNF treatment decreases inflammation during progressive EAE. **a** Total numbers of CD45^hi^ inflammatory cells in the CNS at d30 p.i. of WT and GFAPγR1∆ mice treated with α-TNF or α-βgal mAb. **b** Frequencies of CD8^+^ and CD4^+^ T cells as well as CD11b^+^ myeloid cells within CD45^hi^ infiltrating cells in the CNS of the three mouse groups as depicted. **c** Total numbers of Th1 (IFN-γ-producing) and Th17 (IL-17 producing) CD4^+^ T cells derived from the CNS (d30 p.i.) assessed after PMA and ionomycin stimulation. **d** IFN-γ and IL-17 in supernatants of splenocytes from immunized mice (d30 p.i.) stimulated with MOG^35–55^ peptide as determined by ELISA. Data represent the mean values and ± SEM of three separate experiments with five mice per group per experiment. *P* values determined by Student’s *t* test

To determine if TNF also contributed to sustained CNS immune cell infiltration during progressive EAE, CNS inflammatory cells in anti-TNF treated GFAPγR1∆ were compared to control treated and WT mice by flow cytometry. TNF blockade in GFAPγR1∆ mice indeed reduced inflammatory cells to approximately the level in WT mice with resolving EAE (Fig. 4a). However, similar frequencies of CD4^+^ T cells, CD8^+^ T cells, and macrophages within the inflammatory population (Fig. 4b) suggested a global anti-inflammatory effect, rather than specific effect on macrophages. Effector T cells are primary mediators of disability during EAE [43–45] and both Th1 and Th17 cells are increased in the CNS of GFAPγR1∆ mice during disease progression relative to WT mice [24, 25]. To determine whether TNF blockade preferentially reduced a specific subset, thereby altering the ratio of Th1 and Th17 cells, we assessed the number of CD4^+^ T cells producing IFN-γ or IL-17, respectively. Anti-TNF treatment reduced both effector Th1 and Th17 cells to a similar extent approximating the numbers in WT mice (Fig. 4c). These data are consistent with an unbiased reduction in CNS inflammatory cells, confirming TNF as a critical pro-inflammatory cytokine promoting the maintenance of effector T cells within the CNS.

Uncontrolled CNS inflammatory responses may be driven by ongoing peripheral immune activation [46–49]. We therefore examined the effect of TNF blockade on peripheral effector T cell responses in GFAPγR1∆ mice by measuring MOG^35-55^-specific cytokine secretion. Splenic T cells from GFAPγR1∆ mice with progressive EAE secreted increased IFN-γ and IL-17 compared to WT with EAE. Importantly however, anti-TNF treatment did not reduce the levels of either T cell-produced cytokine in splenic T cells (Fig. 4d). Similar results were obtained following analysis of T cells derived from cervical lymph nodes (data not shown).

### TNF affects blood brain barrier integrity

**Fig. 5:**
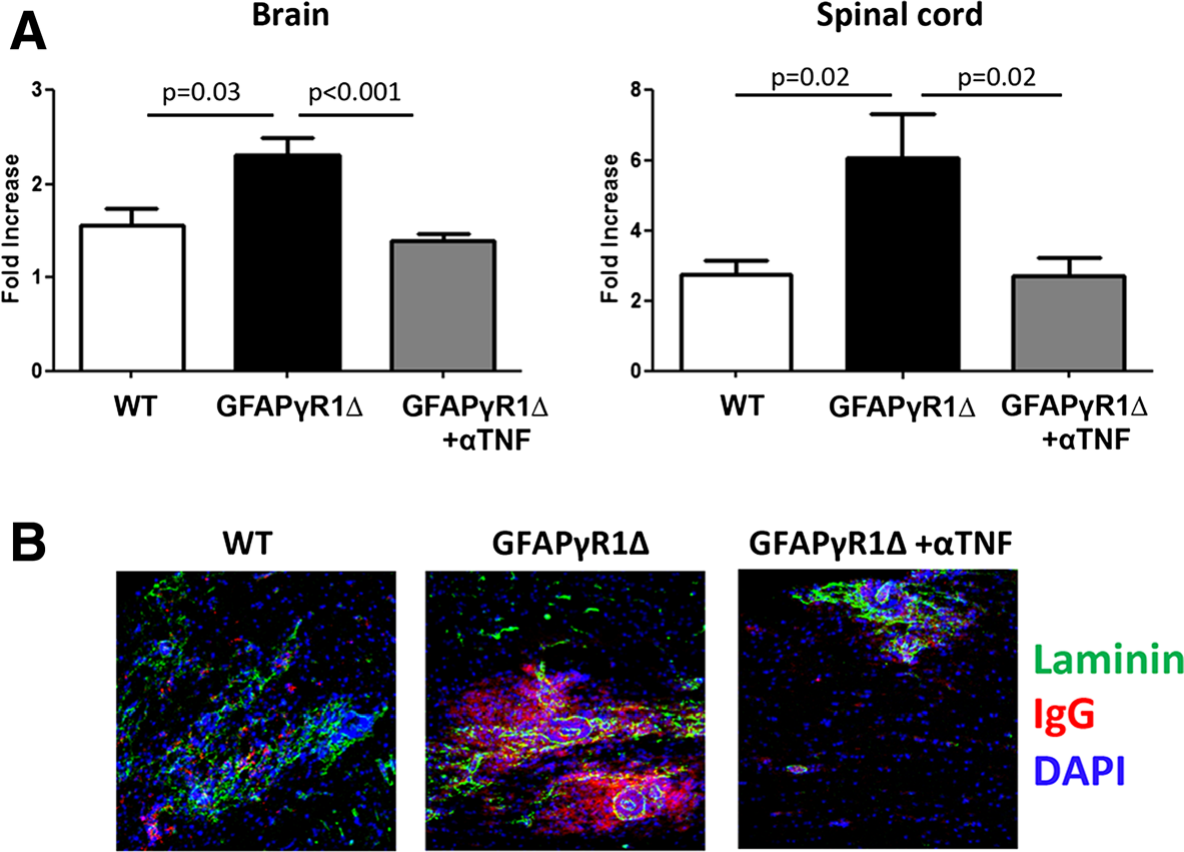
TNF blockade restores BBB integrity during progressive EAE. **a** BBB integrity in brains and spinal cords determined by sodium fluorescein permeability assay determined at day 25 p.i. in WT and GFAPγR1∆ mice treated with α-TNF or control mAb. Data is represented as fold increase over naïve values with six mice per group. **b** Confocal images (Z-stack, magnification ×40) of spinal cords stained with laminin (*green*), IgG (*red*), and DAPI (*blue*) at d30 p.i. Images representative of two individual mice per group. *P* values determined by Student’s *t* test

Among its many pleiotropic functions, TNF increases BBB permeability by disrupting tight junctions [46–49]. The finding that TNF blockade did not reduce peripheral self-reactive effector T cells suggested that TNF influenced CNS inflammation via altering BBB integrity. Both brains and spinal cords of GFAPγR1∆ with progressive EAE exhibited increased BBB permeability compared to WT mice with resolving EAE (Fig. 5a). Anti-TNF treatment restored BBB integrity in GFAPγR1∆ to that exhibited by WT mice. Restoration of BBB integrity by TNF blockade in the spinal cord was confirmed by immunohistochemistry (Fig. 5b). Staining for laminin, IgG, and nuclei in spinal cords from WT mice with resolving EAE showed most serum IgG contained within blood vessels, whereas serum IgG had clearly leaked into the parenchyma in GFAPγR1∆ mice. Spinal cords from GFAPγR1∆ treated with anti-TNF exhibited reduced serum IgG leakage into the spinal cord parenchyma. These data support the concept that ongoing BBB disruption contributes to progressive EAE and that one aspect of protection by TNF blockade is restoration of BBB integrity.

### TNF blockade promotes anti-inflammatory responses

**Fig. 6:**
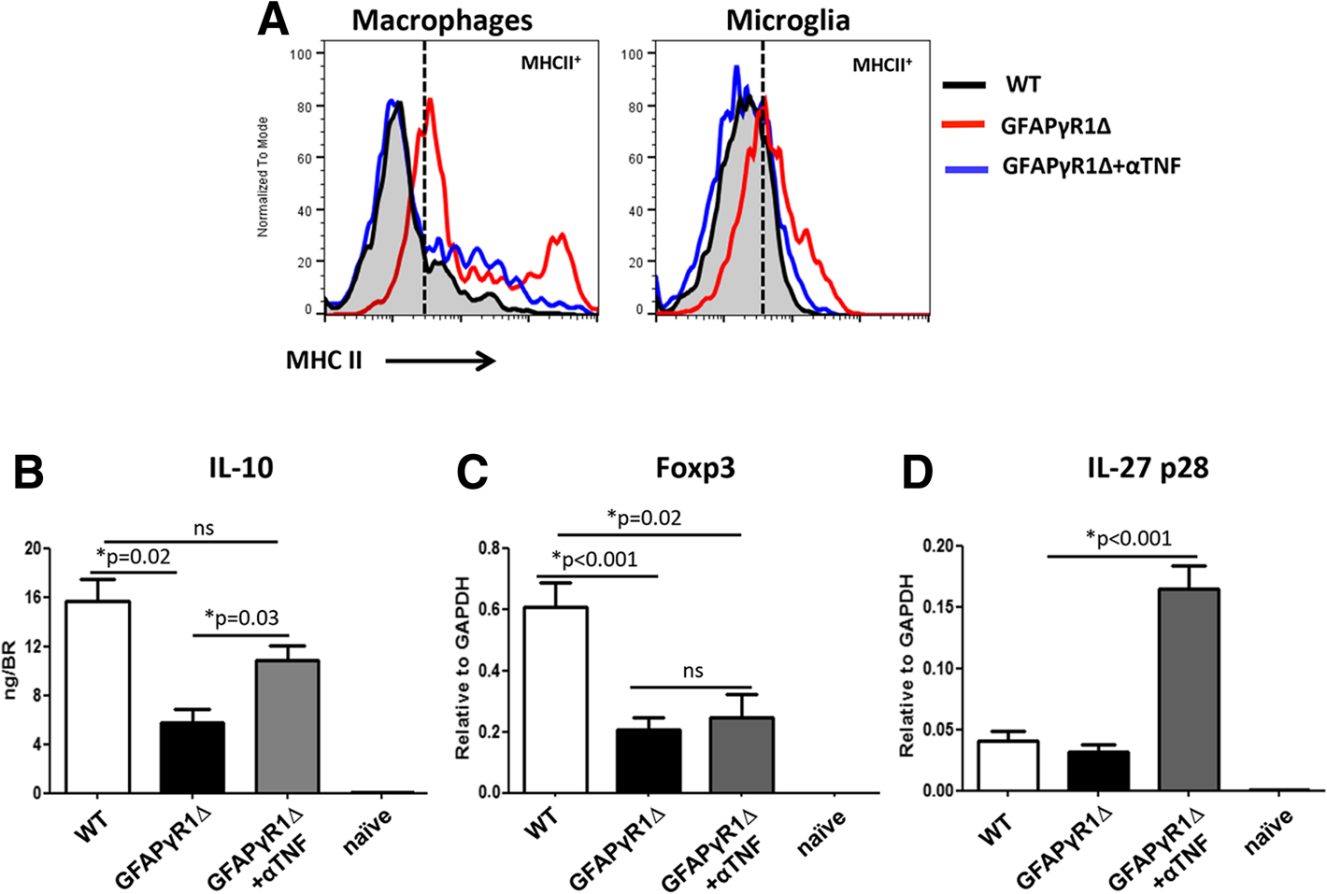
TNF neutralization promotes anti-inflammatory responses. **a** MHC class II expression on macrophages and microglia derived from WT and GFAPγR1∆ mice treated with α-TNF or control mAb determined by flow cytometry at d30p.i. **b** IL-10 levels in the CNS of mouse groups as depicted determined by ELISA (D30p.i.). Foxp3 (**c**) and IL-27p28 (**d**) mRNA expression in the CNS determined by RT-PCR. Data represent the mean values and ± SEM of three separate experiments with five mice per group per experiment. *P* values determined by Student’s *t* test

TNF-induced activation of macrophages and microglia in vitro and in vivo [36, 50–53] is also a hallmark of progressive EAE in GFAPγR1∆ mice [24, 25]. MHC class II expression was evaluated as an activation marker to verify that TNF blockade reduces macrophage and microglial activation. Elevated MHC class II expression on macrophages and to a lesser extent on microglia within the CNS of GFAPγR1∆ compared to WT mice [24, 25] was indeed counteracted by anti-TNF treatment (Fig. 6a). Reduced MHC class II expression on both the infiltrating macrophages and microglia implied reduced activation and antigen presenting capacity of macrophages, thereby further limiting sustained inflammation.

The promotion of anti-inflammatory factors, particularly IL-10, may provide an additional mechanism by which TNF blockade diminishes effector T cell function. This notion is consistent with decreased IL-10, an inflammatory regulator limiting MHC class II expression on antigen presenting cells [54, 55], in the CNS of GFAPγR1∆ mice with progressive EAE. TNF blockade did elevate IL-10 within the CNS. Although the rwofold increase was significant, it did not reach levels found in WT mice undergoing disease remission (Fig. 6b). Primary sources of IL-10 during EAE include Foxp3^+^ T regulatory cells (Treg) and the IL-27-dependent antigen specific T regulatory (Tr-1) cells [56–59]. As overall numbers of CD4^+^ T cells, including Th1 and Th17 cells were reduced by anti-TNF treatment, increased IL-10 suggested a more prominent function of either regulatory cell type. Both Foxp3 mRNA levels (Fig. 6c) as well as Foxp3^+^ CD4^+^ T cells (data not shown) were similar in anti-TNF treated GFAPγR1∆ mice and controls, confirming a relative increase compared to pathogenic effector T cells. Transcript levels for IL-27p28, required to promote Tr-1 cells, was even increased ~3-fold (Fig. 6d), suggesting that TNF may contribute to progressive disease via suppressing Tr-1 cells in addition to sustaining MHC class II expression.

## Discussion

Despite preclinical data strongly supporting beneficial effects of suppressing TNF, TNF blockade in MS patients has shown variable success. For example, treating relapsing-remitting patients with a soluble TNFR1 fusion protein that neutralizes TNF increased the relapse rate and neurological deficits [15]. Similarly, increased lesion numbers and lymphocytes following anti-TNF mAb treatment in two patients with rapidly progressing MS was consistent with disease exacerbation [14]. By contrast, treatment of progressive MS patients with the TNF inhibiting drug pirfenidone reduced the incidence of relapses [31, 32]. The ineffectiveness of anti-TNF immune therapy in acute and progressive MS likely reflects the divergent roles of downstream signaling via the two TNF receptors as well as the clinical state or phase during treatment. This concept is supported by dampened disease severity following TNF blockade during the early acute phase of EAE, when TNF primarily exerts effects through TNFR1. By contrast, as the local inflammatory response including TNF expression evolves, thereby inducing TNFR2 expression, TNF blockade also abrogates protective TNFR2 signaling events that both induce remyelination and immunosuppressive action [18, 19]. Although the induction of nerve growth factor (NGF), which can suppress TNF/TNFR1 signaling, has been implicated in anti-inflammatory effects of TNF/TNFR2 signaling in promoting remyelination [60–62], analysis of NGF mRNA induction in spinal cords did not reveal significant differences following anti-TNF treatment in mice undergoing progressive EAE (data not shown). NGF mRNA levels were increased ~6-fold in both treated and control treated groups relative to naïve mice at day 30 p.i. Furthermore, analysis of mRNA expression for brain-derived neurotropic factor (BDNF), also implicated in promoting remyelination [4, 60, 63], revealed no induction at day 30 post EAE induction in spinal cords of GFAPγR1∆ mice treated or not with anti-TNF Ab relative naïve mice (data not shown). Our data thus demonstrate that TNF blockade during a sustained acute disease, as reflected in the pathogenesis of progressive MS, results in a diminution of both clinical disability and lesion progression independent of NGF or BDNF mRNA alterations.

Activation-induced production of TNF by numerous cell types further contributes to the complex regulation of TNF and distinct outcomes of intervention. Both microglia/macrophages and astrocytes can secrete TNF in vitro and in vivo, yet little is known on the regulation in vivo [40, 41]. In MS, TNF is associated with both astrocytes and foamy macrophages at the edge and center of the lesion, respectively [2]. In vitro stimulation of astrocytes with lipopolysaccharide and IFN-γ induces TNF production, whereas IFN-γ stimulation alone does not [41]. However, astrocytes purified from dual transgenic GFAPγR1∆/GFAP-GFP reporter mice during progressive EAE expressed minimal TNF mRNA. This was confirmed by the inability to detect TNF production by astrocytes in vivo either in WT mice during acute and post-acute EAE or in mice exhibiting progressive EAE. There was also no evidence for T cell-mediated TNF production in vivo. Our data rather confirmed that TNF is primarily produced by both infiltrating macrophages and microglia during acute MOG induced EAE [37]. Similarly, GFAP∆R1γ mice displayed no alterations in the cellular TNF pattern during acute disease, although TNF^+^ cells were slightly increased (Fig. 1). Surprisingly, while TNF production was considerably reduced by both macrophages and microglia during the recovery phase in WT mice, TNF production was specifically sustained in CNS infiltrating macrophages, but not microglia, during progressive EAE. Together, our results suggest that the sustained clinical disease, disability, increased mortality, and increasing tissue damage associated with progressive disease are all associated with TNF production by the infiltrating macrophage population and not microglia. These are the first data demonstrating differential regulation of TNF expression in macrophages versus microglia in an environment of sustained inflammation established by the inability of astrocytes to respond to IFN-γ. While these results support an astrocyte dependent component in specifically affecting ongoing TNF expression by bone marrow derived macrophages, the underlying molecular mechanisms remain to be resolved. A better understanding of the relative expression of soluble versus membrane bound TNF as well as the balance between TNFR1 versus TNFR2 engagement on distinct CNS cell types may provide clues. We can also not exclude dysregulation of the TNF, NGF, and BDNF signaling triad in contributing to progressive disease [60], as signaling not only of TNF but also neurotropic factors may be altered.

The protective effect of anti-TNF Ab treatment on progressive EAE supported that macrophage-derived TNF is a detrimental contributor to disability. Improved clinical disease and survival directly coincided with diminished cellular CNS inflammation and demyelination. Interestingly, TNF blockade decreased the overall numbers of CNS infiltrating immune cells, without affecting a specific population or the ratio of Th1 and Th17 cells. A potential therapeutic mechanism by which TNF blockade reduces CNS inflammation during progressive EAE is the regulation of peripheral immune response, similar to the effects described during induction of acute EAE [64]. However, TNF blockade did not reduce increased self-reactive Th1 and Th17 responses in the periphery, suggesting that the primary site of detrimental TNF activity is within the CNS. A critical anatomical site affected by TNF is the BBB, which is not only is essential to maintain CNS homeostasis [65] but also limits CNS access of peripheral inflammatory cells [66]. TNF is implicated in disruption of the BBB in several models of CNS inflammation [46–49, 67, 68]. In EAE, TNF disrupts the BBB by altering tight junctions [46–49], thereby promoting the influx of inflammatory cells into the CNS parenchyma. Moreover, TNF^-/-^ mice have reduced BBB permeability compared to WT mice during EAE [67]. Consistent with these reports, decreased infiltration of immune cells by TNF blockade during progressive EAE correlated with prevention of further BBB breakdown noted in untreated mice. In this context, it is interesting to note that TNF activates IL-6 production and signaling in human primary endothelial cells in vitro, which synergizes with TNF to promote permeabilization [69]. IL-6 blockade during progressive EAE also ameliorated disease progression, but to a lesser extent than anti-TNF treatment [25], supporting a more prominent role of TNF. Whether control of BBB breakdown was the primary mechanism by which TNF blockade ameliorated progressive CNS pathology remains unclear. Increased T cell proliferation in the CNS during progressive EAE [25] may reflect facilitated recruitment of peripherally activated cells resuming proliferation in the CNS. Alternatively, TNF may drive local proliferation independent of increased BBB permeability.

An additional mechanism associated with the failure to transition from acute autoimmune mediated-inflammation towards remission in progressive EAE is dysregulation of IL-10, a potent regulator of T cell activation [54, 55, 70]. IL-10, produced primarily by Treg and Tr1 cells during EAE [71], is decreased during progressive EAE in GFAPγR1∆ mice [24]. Furthermore, IL-10^-/-^ mice are susceptible to severe EAE, whereas IL-10 administration suppresses acute disease [57, 71, 72]. TNF blockade indeed increased IL-10 expression within the CNS concomitant with upregulation of IL-27 required for the conversion of antigen specific CD4^+^ T cells into IL-10 secreting Tr1 cells [57, 58, 73–75]. By contrast, frequencies of Treg within the CNS remained unaltered. Unchecked TNF may thus counteract establishment of an anti-inflammatory milieu by limiting IL-27 expression and suppressing the induction of Tr1 cells. In a viral-induced demyelination model, IL-10 controls demyelination by limiting both the size and number of demyelinating lesions [76]. GFAPγR1∆ mice exhibit increased demyelination during EAE, which correlates with decreased IL-10 levels [24, 25]. Interestingly, anti-TNF treatment also significantly reduced demyelination in GFAPγR1∆ mice, suggesting increased IL-10 may contribute to controlling demyelination during progressive EAE after anti-TNF treatment.

TNF has also been implicated in promoting macrophage polarization to a pro-inflammatory M1 phenotype following spinal cord injury, thereby contributing to tissue damage [36]. Using MHC class II expression as a readout, TNF blockade decreased macrophage and to a lesser extent microglia activation. While this supported a paracrine effect of sustained macrophage production of TNF on promoting microglial activation, the cause and effect of simultaneously suppressed IL-10, which also reduces MHC class II expression [54, 55, 70] remains to be elucidated. Furthermore, as TNF is initially produced as transmembrane TNF, neither histological evaluation nor intracellular flow cytometry can distinguish between the potential contributions of soluble TNF/TNFR1 versus transmembrane TNF/TNFR2 interactions to pathogenesis or disease resolution. Sustained TNF in the absence of IFN-γ signaling to astrocytes may alter the status of protective TNFR2 expression on glia or infiltrating macrophages. In WT mice undergoing EAE, clinical conversion to remission may be associated with preferential TNFR2 expression and/or membrane bound TNF. This pathway may be dysregulated in progressive EAE by enhanced proteolytic activity resulting in soluble TNF.

## Conclusions

Our data demonstrate that sustained TNF production by CNS infiltrating macrophages promotes BBB permeability, inflammation, and demyelination, thereby progressively increasing disability and morbidity. While our studies confirmed the failure of anti-TNF therapy in WT mice undergoing EAE [13, 22, 23], blockade of sustained TNF ameliorated both disease severity and limited ongoing demyelination during progressive EAE. Importantly, our results are reminiscent of studies demonstrating a reduction in relapses and improved disability with only mild secondary effects in secondary progressive MS patients treated with the TNF inhibitor pirfenidone [31, 32]. Together, these data support a therapeutic approach to limiting TNF as a potential treatment to slow down or halt progressive forms of MS.

## Abbreviations

BBB: blood brain barrier
CNS: central nervous system
EAE: experimental autoimmune encephalomyelitis
GFAP: glial fibrillary acidic protein
GFP: green fluorescent protein
i.p.: intra peritoneal
IFN-γ: interferon-γ
IL: interleukin
LFB: luxol fast blue
mAb: monoclonal antibody
MFI: mean fluorescent intensity
MHC: major histocompatibility complex
MOG: myelin oligodendrocyte glycoprotein
MS: multiple sclerosis
NaF: sodium fluorescein
p.i.: post-immunization
PMA: phorbol 12-myristate 13-acetate
TNF: Tumor necrosis factor
Treg: regulatory T cell
WT: wild type
β-gal: β-galactosidase

## References

[1] McCoy MK, Tansey MG (2008). TNF signaling inhibition in the CNS: implications for normal brain function and neurodegenerative disease. J Neuroinflammation.

[2] Selmaj K, Raine CS, Cannella B, Brosnan CF (1991). Identification of lymphotoxin and tumor necrosis factor in multiple sclerosis lesions. J Clin Invest.

[3] Spuler S, Yousry T, Scheller A, Voltz R, Holler E, Hartmann M, Wick M, Hohlfeld R (1996). Multiple sclerosis: prospective analysis of TNF-alpha and 55 kDa TNF receptor in CSF and serum in correlation with clinical and MRI activity. J Neuroimmunol.

[4] Begum F, Zhu W, Cortes C, MacNeil B, Namaka M (2013). Elevation of tumor necrosis factor alpha in dorsal root ganglia and spinal cord is associated with neuroimmune modulation of pain in an animal model of multiple sclerosis. J Neuroimmune Pharmacol.

[5] Probert L, Akassoglou K, Pasparakis M, Kontogeorgos G, Kollias G (1995). Spontaneous inflammatory demyelinating disease in transgenic mice showing central nervous system-specific expression of tumor necrosis factor alpha. Proc Natl Acad Sci U S A.

[6] Akassoglou K, Probert L, Kontogeorgos G, Kollias G (1997). Astrocyte-specific but not neuron-specific transmembrane TNF triggers inflammation and degeneration in the central nervous system of transgenic mice. J Immunol.

[7] Dal Canto RA, Shaw MK, Nolan GP, Steinman L, Fathman CG (1999). Local delivery of TNF by retrovirus-transduced T lymphocytes exacerbates experimental autoimmune encephalomyelitis. Clin Immunol.

[8] Selmaj KW, Raine CS (1988). Tumor necrosis factor mediates myelin and oligodendrocyte damage in vitro. Ann Neurol.

[9] Selmaj K, Raine CS (1988). Tumor necrosis factor mediates myelin damage in organotypic cultures of nervous tissue. Ann N Y Acad Sci.

[10] Hisahara S, Shoji S, Okano H, Miura M (1997). ICE/CED-3 family executes oligodendrocyte apoptosis by tumor necrosis factor. J Neurochem.

[11] Korn T, Magnus T, Jung S (2005). Autoantigen specific T cells inhibit glutamate uptake in astrocytes by decreasing expression of astrocytic glutamate transporter GLAST: a mechanism mediated by tumor necrosis factor-alpha. FASEB J.

[12] Ruddle NH, Bergman CM, McGrath KM, Lingenheld EG, Grunnet ML, Padula SJ, Clark RB (1990). An antibody to lymphotoxin and tumor necrosis factor prevents transfer of experimental allergic encephalomyelitis. J Exp Med.

[13] Baker D, Butler D, Scallon BJ, O’Neill JK, Turk JL, Feldmann M (1994). Control of established experimental allergic encephalomyelitis by inhibition of tumor necrosis factor (TNF) activity within the central nervous system using monoclonal antibodies and TNF receptor-immunoglobulin fusion proteins. Eur J Immunol.

[14] van Oosten BW, Barkhof F, Truyen L, Boringa JB, Bertelsmann FW, von Blomberg BM, Woody JN, Hartung HP, Polman CH (1996). Increased MRI activity and immune activation in two multiple sclerosis patients treated with the monoclonal anti-tumor necrosis factor antibody cA2. Neurology.

[15] TNF neutralization in MS: results of a randomized, placebo-controlled multicenter study. The Lenercept Multiple Sclerosis Study Group and the University of British Columbia MS/MRI Analysis Group. Neurology. 1999; 53:457-65. 10449104

[16] Holtmann MH, Neurath MF (2004). Differential TNF-signaling in chronic inflammatory disorders. Curr Mol Med.

[17] Alexopoulou L, Kranidioti K, Xanthoulea S, Denis M, Kotanidou A, Douni E, Blackshear PJ, Kontoyiannis DL, Kollias G (2006). Transmembrane TNF protects mutant mice against intracellular bacterial infections, chronic inflammation and autoimmunity. Eur J Immunol.

[18] Arnett HA, Mason J, Marino M, Suzuki K, Matsushima GK, Ting JP (2001). TNF alpha promotes proliferation of oligodendrocyte progenitors and remyelination. Nat Neurosci.

[19] Kassiotis G, Kollias G (2001). Uncoupling the proinflammatory from the immunosuppressive properties of tumor necrosis factor (TNF) at the p55 TNF receptor level: implications for pathogenesis and therapy of autoimmune demyelination. J Exp Med.

[20] Eugster HP, Frei K, Bachmann R, Bluethmann H, Lassmann H, Fontana A (1999). Severity of symptoms and demyelination in MOG-induced EAE depends on TNFR1. Eur J Immunol.

[21] Liu J, Marino MW, Wong G, Grail D, Dunn A, Bettadapura J, Slavin AJ, Old L, Bernard CC (1998). TNF is a potent anti-inflammatory cytokine in autoimmune-mediated demyelination. Nat Med.

[22] Brambilla R, Ashbaugh JJ, Magliozzi R, Dellarole A, Karmally S, Szymkowski DE, Bethea JR (2011). Inhibition of soluble tumour necrosis factor is therapeutic in experimental autoimmune encephalomyelitis and promotes axon preservation and remyelination. Brain.

[23] Batoulis H, Recks MS, Holland FO, Thomalla F, Williams RO, Kuerten S (2014). Blockade of tumour necrosis factor-alpha in experimental autoimmune encephalomyelitis reveals differential effects on the antigen-specific immune response and central nervous system histopathology. Clin Exp Immunol.

[24] Hindinger C, Bergmann CC, Hinton DR, Phares TW, Parra GI, Hussain S, Savarin C, Atkinson RD, Stohlman SA (2012). IFN-gamma signaling to astrocytes protects from autoimmune mediated neurological disability. PLoS One.

[25] Savarin C, Hinton DR, Valentin-Torres A, Chen Z, Trapp BD, Bergmann CC, Stohlman SA (2015). Astrocyte response to IFN-gamma limits IL-6-mediated microglia activation and progressive autoimmune encephalomyelitis. J Neuroinflammation.

[26] Gijbels K, Brocke S, Abrams JS, Steinman L (1995). Administration of neutralizing antibodies to interleukin-6 (IL-6) reduces experimental autoimmune encephalomyelitis and is associated with elevated levels of IL-6 bioactivity in central nervous system and circulation. Mol Med.

[27] Serada S, Fujimoto M, Mihara M, Koike N, Ohsugi Y, Nomura S, Yoshida H, Nishikawa T, Terabe F, Ohkawara T (2008). IL-6 blockade inhibits the induction of myelin antigen-specific Th17 cells and Th1 cells in experimental autoimmune encephalomyelitis. Proc Natl Acad Sci U S A.

[28] Minogue AM, Barrett JP, Lynch MA (2012). LPS-induced release of IL-6 from glia modulates production of IL-1beta in a JAK2-dependent manner. J Neuroinflammation.

[29] Benveniste EN, Sparacio SM, Norris JG, Grenett HE, Fuller GM (1990). Induction and regulation of interleukin-6 gene expression in rat astrocytes. J Neuroimmunol.

[30] Van Wagoner NJ, Oh JW, Repovic P, Benveniste EN (1999). Interleukin-6 (IL-6) production by astrocytes: autocrine regulation by IL-6 and the soluble IL-6 receptor. J Neurosci.

[31] Walker JE, Margolin SB (2001). Pirfenidone for chronic progressive multiple sclerosis. Mult Scler.

[32] Walker JE, Giri SN, Margolin SB (2005). A double-blind, randomized, controlled study of oral pirfenidone for treatment of secondary progressive multiple sclerosis. Mult Scler.

[33] Hindinger C, Gonzalez JM, Bergmann CC, Fuss B, Hinton DR, Atkinson RD, Macklin WB, Stohlman SA (2005). Astrocyte expression of a dominant-negative interferon-gamma receptor. J Neurosci Res.

[34] Kapil P, Stohlman SA, Hinton DR, Bergmann CC (2014). PKR mediated regulation of inflammation and IL-10 during viral encephalomyelitis. J Neuroimmunol.

[35] Phares TW, Kean RB, Mikheeva T, Hooper DC (2006). Regional differences in blood-brain barrier permeability changes and inflammation in the apathogenic clearance of virus from the central nervous system. J Immunol.

[36] Kroner A, Greenhalgh AD, Zarruk JG, Passos Dos Santos R, Gaestel M, David S (2014). TNF and increased intracellular iron alter macrophage polarization to a detrimental M1 phenotype in the injured spinal cord. Neuron.

[37] Renno T, Krakowski M, Piccirillo C, Lin JY, Owens T (1995). TNF-alpha expression by resident microglia and infiltrating leukocytes in the central nervous system of mice with experimental allergic encephalomyelitis. Regulation by Th1 cytokines. J Immunol.

[38] Begolka WS, Vanderlugt CL, Rahbe SM, Miller SD (1998). Differential expression of inflammatory cytokines parallels progression of central nervous system pathology in two clinically distinct models of multiple sclerosis. J Immunol.

[39] Zorzella-Pezavento SF, Chiuso-Minicucci F, Franca TG, Ishikawa LL, da Rosa LC, Marques C, Ikoma MR, Sartori A (2013). Persistent inflammation in the CNS during chronic EAE despite local absence of IL-17 production. Mediators Inflamm.

[40] Chung IY, Benveniste EN (1990). Tumor necrosis factor-alpha production by astrocytes. Induction by lipopolysaccharide, IFN-gamma, and IL-1 beta. J Immunol.

[41] Chung IY, Norris JG, Benveniste EN (1991). Differential tumor necrosis factor alpha expression by astrocytes from experimental allergic encephalomyelitis-susceptible and -resistant rat strains. J Exp Med.

[42] Murray RZ, Kay JG, Sangermani DG, Stow JL (2005). A role for the phagosome in cytokine secretion. Science.

[43] Fletcher JM, Lalor SJ, Sweeney CM, Tubridy N, Mills KH (2010). T cells in multiple sclerosis and experimental autoimmune encephalomyelitis. Clin Exp Immunol.

[44] Codarri L, Gyulveszi G, Tosevski V, Hesske L, Fontana A, Magnenat L, Suter T, Becher B (2011). RORgammat drives production of the cytokine GM-CSF in helper T cells, which is essential for the effector phase of autoimmune neuroinflammation. Nat Immunol.

[45] Ponomarev ED, Shriver LP, Maresz K, Pedras-Vasconcelos J, Verthelyi D, Dittel BN (2007). GM-CSF production by autoreactive T cells is required for the activation of microglial cells and the onset of experimental autoimmune encephalomyelitis. J Immunol.

[46] Tsao N, Hsu HP, Wu CM, Liu CC, Lei HY (2001). Tumour necrosis factor-alpha causes an increase in blood-brain barrier permeability during sepsis. J Med Microbiol.

[47] Tsuge M, Yasui K, Ichiyawa T, Saito Y, Nagaoka Y, Yashiro M, Yamashita N, Morishima T (2010). Increase of tumor necrosis factor-alpha in the blood induces early activation of matrix metalloproteinase-9 in the brain. Microbiol Immunol.

[48] Lv S, Song HL, Zhou Y, Li LX, Cui W, Wang W, Liu P (2010). Tumour necrosis factor-alpha affects blood-brain barrier permeability and tight junction-associated occludin in acute liver failure. Liver Int.

[49] Nishioku T, Matsumoto J, Dohgu S, Sumi N, Miyao K, Takata F, Shuto H, Yamauchi A, Kataoka Y (2010). Tumor necrosis factor-alpha mediates the blood-brain barrier dysfunction induced by activated microglia in mouse brain microvascular endothelial cells. J Pharmacol Sci.

[50] Mace KF, Ehrke MJ, Hori K, Maccubbin DL, Mihich E (1988). Role of tumor necrosis factor in macrophage activation and tumoricidal activity. Cancer Res.

[51] Heise MT, Virgin HW (1995). The T-cell-independent role of gamma interferon and tumor necrosis factor alpha in macrophage activation during murine cytomegalovirus and herpes simplex virus infections. J Virol.

[52] Sriram K, Matheson JM, Benkovic SA, Miller DB, Luster MI, O’Callaghan JP (2006). Deficiency of TNF receptors suppresses microglial activation and alters the susceptibility of brain regions to MPTP-induced neurotoxicity: role of TNF-alpha. FASEB J.

[53] Kuno R, Wang J, Kawanokuchi J, Takeuchi H, Mizuno T, Suzumura A (2005). Autocrine activation of microglia by tumor necrosis factor-alpha. J Neuroimmunol.

[54] Chadban SJ, Tesch GH, Foti R, Lan HY, Atkins RC, Nikolic-Paterson DJ (1998). Interleukin-10 differentially modulates MHC class II expression by mesangial cells and macrophages in vitro and in vivo. Immunology.

[55] Koppelman B, Neefjes JJ, de Vries JE, de Waal Malefyt R (1997). Interleukin-10 down-regulates MHC class II alpha beta peptide complexes at the plasma membrane of monocytes by affecting arrival and recycling. Immunity.

[56] Huss DJ, Winger RC, Peng H, Yang Y, Racke MK, Lovett-Racke AE (2010). TGF-beta enhances effector Th1 cell activation but promotes self-regulation via IL-10. J Immunol.

[57] Fitzgerald DC, Zhang GX, El-Behi M, Fonseca-Kelly Z, Li H, Yu S, Saris CJ, Gran B, Ciric B, Rostami A (2007). Suppression of autoimmune inflammation of the central nervous system by interleukin 10 secreted by interleukin 27-stimulated T cells. Nat Immunol.

[58] Stumhofer JS, Silver JS, Laurence A, Porrett PM, Harris TH, Turka LA, Ernst M, Saris CJ, O’Shea JJ, Hunter CA (2007). Interleukins 27 and 6 induce STAT3-mediated T cell production of interleukin 10. Nat Immunol.

[59] Korn T, Reddy J, Gao W, Bettelli E, Awasthi A, Petersen TR, Backstrom BT, Sobel RA, Wucherpfennig KW, Strom TB (2007). Myelin-specific regulatory T cells accumulate in the CNS but fail to control autoimmune inflammation. Nat Med.

[60] KhorshidAhmad T, Acosta C, Cortes C, Lakowski TM, Gangadaran S, Namaka M (2016). Transcriptional regulation of brain-derived neurotrophic factor (BDNF) by methyl CpG binding protein 2 (MeCP2): a novel mechanism for re-myelination and/or myelin repair involved in the treatment of multiple sclerosis (MS). Mol Neurobiol.

[61] Wheeler MA, Heffner DL, Kim S, Espy SM, Spano AJ, Cleland CL, Deppmann CD (2014). TNF-alpha/TNFR1 signaling is required for the development and function of primary nociceptors. Neuron.

[62] Takei Y, Laskey R (2008). Tumor necrosis factor alpha regulates responses to nerve growth factor, promoting neural cell survival but suppressing differentiation of neuroblastoma cells. Mol Biol Cell.

[63] Melanson M, Miao P, Eisenstat D, Gong Y, Gu X, Au K, Zhu W, Begum F, Frost EE, Namaka M (2009). Experimental autoimmune encephalomyelitis-induced upregulation of tumor necrosis factor-alpha in the dorsal root ganglia. Mult Scler.

[64] Cserr HF, Knopf PM (1992). Cervical lymphatics, the blood-brain barrier and the immunoreactivity of the brain: a new view. Immunol Today.

[65] Weiss N, Miller F, Cazaubon S, Couraud PO (2009). The blood-brain barrier in brain homeostasis and neurological diseases. Biochim Biophys Acta.

[66] Larochelle C, Alvarez JI, Prat A (2011). How do immune cells overcome the blood-brain barrier in multiple sclerosis?. FEBS Lett.

[67] Fabis MJ, Scott GS, Kean RB, Koprowski H, Hooper DC (2007). Loss of blood-brain barrier integrity in the spinal cord is common to experimental allergic encephalomyelitis in knockout mouse models. Proc Natl Acad Sci U S A.

[68] Zhao C, Ling Z, Newman MB, Bhatia A, Carvey PM (2007). TNF-alpha knockout and minocycline treatment attenuates blood-brain barrier leakage in MPTP-treated mice. Neurobiol Dis.

[69] Rochfort KD, Collins LE, McLoughlin A, Cummins PM. TNF-alpha-mediated disruption of cerebrovascular endothelial barrier integrity in vitro involves the production of proinflammatory IL-6. J Neurochem. 2015. 10.1111/jnc.13408 26499872

[70] Thibodeau J, Bourgeois-Daigneault MC, Huppe G, Tremblay J, Aumont A, Houde M, Bartee E, Brunet A, Gauvreau ME, de Gassart A (2008). Interleukin-10-induced MARCH1 mediates intracellular sequestration of MHC class II in monocytes. Eur J Immunol.

[71] Zhang X, Koldzic DN, Izikson L, Reddy J, Nazareno RF, Sakaguchi S, Kuchroo VK, Weiner HL (2004). IL-10 is involved in the suppression of experimental autoimmune encephalomyelitis by CD25 + CD4+ regulatory T cells. Int Immunol.

[72] Bettelli E, Das MP, Howard ED, Weiner HL, Sobel RA, Kuchroo VK (1998). IL-10 is critical in the regulation of autoimmune encephalomyelitis as demonstrated by studies of IL-10- and IL-4-deficient and transgenic mice. J Immunol.

[73] Awasthi A, Carrier Y, Peron JP, Bettelli E, Kamanaka M, Flavell RA, Kuchroo VK, Oukka M, Weiner HL (2007). A dominant function for interleukin 27 in generating interleukin 10-producing anti-inflammatory T cells. Nat Immunol.

[74] Pot C, Jin H, Awasthi A, Liu SM, Lai CY, Madan R, Sharpe AH, Karp CL, Miaw SC, Ho IC, Kuchroo VK (2009). Cutting edge: IL-27 induces the transcription factor c-Maf, cytokine IL-21, and the costimulatory receptor ICOS that coordinately act together to promote differentiation of IL-10-producing Tr1 cells. J Immunol.

[75] Apetoh L, Quintana FJ, Pot C, Joller N, Xiao S, Kumar D, Burns EJ, Sherr DH, Weiner HL, Kuchroo VK (2010). The aryl hydrocarbon receptor interacts with c-Maf to promote the differentiation of type 1 regulatory T cells induced by IL-27. Nat Immunol.

[76] Puntambekar SS, Hinton DR, Yin X, Savarin C, Bergmann CC, Trapp BD, et al. Interleukin-10 is a critical regulator of white matter lesion containment following viral induced demyelination. Glia. 2015. 10.1002/glia.22880 PMC4755156 26132901

